# Serum Uric Acid Trajectories in Multiple Sclerosis: A 5-Year Longitudinal Comparison of High- and Moderate-Efficacy Therapies

**DOI:** 10.3390/medsci14020256

**Published:** 2026-05-15

**Authors:** Laura-Elena Cucu, Alina Săcărescu, Andra Oancea, Bogdan Emilian Ignat, Cristina Grosu, Laura-Cristina Baciu, Costin Chirica, Gabriela Popescu, Alexandra Maștaleru, Maria-Magdalena Leon

**Affiliations:** 1 Doctoral School, Grigore T. Popa University of Medicine and Pharmacy, 700115 Iasi, Romania; dudau.laura-elena@d.umfiasi.ro (L.-E.C.);; 2 Clinical Rehabilitation Hospital, 700661 Iasi, Romania; andra.oancea@umfiasi.ro (A.O.); emilian.ignat@umfiasi.ro (B.E.I.); cristina.grosu@umfiasi.ro (C.G.); gabriela.popescu@umfiasi.ro (G.P.); alexandra.mastaleru@umfiasi.ro (A.M.); maria.leon@umfiasi.ro (M.-M.L.); 3 Department of Neurology, Grigore T. Popa University of Medicine and Pharmacy, 700115 Iasi, Romania; 4 Department of Medical Specialties I, Grigore T. Popa University of Medicine and Pharmacy, 700115 Iasi, Romania

**Keywords:** uric acid, multiple sclerosis, disease-modifying therapy, linear mixed models

## Abstract

**Background/Objectives**: Uric acid (UA), a major circulating antioxidant, has been consistently reported at lower levels in multiple sclerosis (MS) patients, yet long-term UA trajectories stratified by disease-modifying therapy (DMT) efficacy remain unexplored. This study aimed to evaluate the 5-year longitudinal evolution of serum UA levels in relapsing-remitting MS (RRMS), comparing high-efficacy (HE-DMT) and moderate-efficacy (ME-DMT) treatment groups. **Methods**: This retrospective longitudinal cohort study included adult RRMS patients who initiated or switched DMTs between January 2020 and June 2025. Serum UA was measured at treatment initiation (T0) and annually for up to 5 years (T1–T5). Linear mixed-effects models adjusted for sex, age, and EDSS were used to examine longitudinal UA trajectories. **Results**: A total of 222 patients were included and followed for up to 5 years across HE-DMT and ME-DMT treatment groups. Groups were comparable at baseline regarding serum UA levels despite significant differences in age, EDSS, and therapy status. Linear mixed-effects modeling demonstrated that serum UA levels changed significantly over time (F(5, 307.04) = 11.03, *p* < 0.01), with a significant main effect of treatment type (F(1, 378.31) = 5.25, *p* = 0.02) and a significant time × treatment interaction (F(5, 307.05) = 2.42, *p* = 0.04), indicating that UA trajectories differed between groups across the follow-up period. In the HE-DMT group, UA levels increased progressively from 4.27 mg/dL at baseline to 5.58 mg/dL at year 4, whereas the ME-DMT group showed an initial decline at year 1 followed by a more gradual increase from 4.19 mg/dL to 5.04 mg/dL at year 4. Sex was a significant independent predictor of UA levels (*p* < 0.01), whereas age and EDSS were not. **Conclusions**: HE-DMTs were associated with an earlier and more pronounced increase in serum UA levels over 5 years compared with ME-DMTs, with distinct trajectories depending on treatment efficacy. These findings suggest that longitudinal UA assessment may serve as a complementary exploratory indicator of the metabolic context associated with DMT efficacy.

## 1. Introduction

Multiple sclerosis (MS) is a chronic inflammatory and neurodegenerative disease of the central nervous system that predominantly affects young adults. Inflammation triggers neurodegenerative processes that can occur independently of demyelination, and involve mitochondrial dysfunction and oxidative stress [1,2]. Reactive nitrogen species such as peroxynitrite contribute to tissue damage and disability progression, making biomarkers of systemic redox status potentially relevant for monitoring disease activity and treatment response [3].

Uric acid (UA) is a major circulating antioxidant and an efficient scavenger of reactive nitrogen species, including peroxynitrite, which has been implicated in MS-related inflammatory tissue injury [4]. Alterations in serum UA levels have been interpreted as reflecting changes in oxidative and nitrosative balance associated with MS-related pathological processes [5,6].

Consistent with this biological role, multiple studies have reported lower serum UA levels in MS patients compared with healthy controls, supporting the concept of altered antioxidant balance in MS [7]. Whereas most previous studies relied on cross-sectional comparisons, a limited number of longitudinal investigations have examined UA variation over time. Moccia et al. reported longitudinal changes in serum UA levels in relapsing-remitting MS (RRMS) patients followed for approximately two years, showing that lower UA values and greater within-patient UA reductions were associated with higher relapse risk, disability progression, and poorer cognitive performance, without stratification by treatment efficacy [8]. Other reports described increases in UA levels in association with specific therapies. For example, a significant rise in serum UA over 12 months was observed in patients treated with glatiramer acetate, whereas interferon-treated patients did not show comparable changes [5]. Similarly, higher UA levels were reported in treated compared with untreated MS patients [9]. Collectively, these studies suggest that UA levels may change over time and under treatment exposure, but do not capture long-term UA trajectories stratified by disease-modifying therapy (DMT) efficacy, leaving uncertain whether long-term changes in UA levels reflect underlying disease activity, treatment-related effects, or their interaction over time.

The present study therefore aimed to evaluate the longitudinal evolution of serum UA levels over 5 years in patients with RRMS, comparing high-efficacy (HE-DMT) and moderate-efficacy (ME-DMT) regimens. We hypothesized that higher treatment potency would be associated with more pronounced restoration of antioxidant balance, reflected in greater and more sustained elevation of serum UA levels in the HE-DMT group compared to the ME-DMT group.

## 2. Materials and Methods

### 2.1. Study Design and Population

This was a retrospective, longitudinal, single-center cohort study.

The study population comprised adult patients diagnosed with RRMS according to the 2017 McDonald criteria [10]. Eligible participants included patients who either initiated their first DMT or switched to a different DMT between January 2020 and June 2025.

Exclusion criteria comprised pregnancy, unclear documentation of clinical dates, known chronic kidney disease or serum creatinine values exceeding 1.5 times the upper limit of the normal reference range, the presence of comorbid rheumatological or other neurological diseases, and a relapse or corticosteroid administration within the preceding 30 days. Additionally, patients receiving antiplatelet agents, diuretics, or UA-lowering therapies at baseline or during follow-up were excluded, as these medications are known to influence serum UA levels [11,12].

### 2.2. Data Collection and Variables

Clinical and laboratory data were extracted from electronic medical records at baseline (T0) and at annual intervals for up to 5 years (T1–T5). To account for real-world scheduling variations, a window of ±3 months was permitted for each annual time point. Baseline (T0) was defined as the point of treatment initiation for treatment-naive patients or the moment of treatment modification for those switching therapies. For the latter, a minimum washout period of 3 months between the previous and current therapies was required prior to the T0 assessment.

DMTs were classified into potency-based categories according to efficacy demonstrated in pivotal clinical trials, following the Delphi Consensus criteria [13]. ME-DMTs included interferons, glatiramer acetate, dimethyl fumarate, and teriflunomide. HE-DMTs included fingolimod, natalizumab, ocrelizumab, ofatumumab, alemtuzumab, and cladribine.

Covariates included in the model were sex, age at treatment initiation (as a continuous variable), disease severity assessed by the Expanded Disability Status Scale (EDSS), and treatment type (HE-DMT versus ME-DMT). These variables were selected based on their known associations with UA metabolism and MS disease course [9,14].

### 2.3. Statistical Analysis

Statistical analyses were performed using JASP (version 0.95.4, Amsterdam, The Netherlands). All tests were two-tailed, and statistical significance was set at *p* < 0.05.

Descriptive statistics were calculated for all baseline variables. Categorical variables (e.g., sex, therapy status) were presented as absolute frequencies (n) and percentages (%) and compared between groups using Fisher’s exact test.

The distribution of continuous variables was assessed for normality. Continuous data with a normal distribution (i.e., UA levels, age) were expressed as mean ± standard deviation (SD) and compared using the independent samples Student’s *t*-test. For variables with a non-normal distribution (i.e., EDSS scores), data were presented as median and interquartile range (IQR) and compared using the Mann–Whitney U test.

Spearman’s rank correlation coefficient was used to assess associations between serum UA levels at treatment initiation (T0) and continuous clinical variables, including age, disease duration, and baseline EDSS score. Additionally, the Mann–Whitney U test was used to compare serum UA levels between sexes at T0.

Linear mixed-effects models were used to examine longitudinal changes in serum UA levels over the follow-up period. This analytical approach utilizes all available data across time points, accounts for within-subject correlation of repeated measurements, and accommodates missing data under the missing-at-random assumption [15]. A random intercept was fitted at the patient level to account for within-patient correlation across therapeutic sequences, including patients contributing more than one sequence.

The dependent variable was serum UA level (mg/dL), measured at treatment initiation or modification (T0) and at yearly intervals thereafter (T1–T5). Time since treatment initiation was included as a categorical variable (T0–T5) to allow for non-linear trajectories. The primary independent variable was treatment type (HE-DMT versus ME-DMT).

The model included the following fixed effects: time, treatment type, sex, age at treatment initiation (continuous), and EDSS score at baseline (continuous). The interaction term between time and treatment type was included to assess whether the trajectory of UA change differed between treatment groups. The model was fitted using restricted maximum likelihood (REML) estimation. Degrees of freedom were calculated using the Satterthwaite approximation.

To examine treatment group differences at specific time points, estimated marginal means and 95% confidence intervals (CIs) were calculated for each treatment group at each time, averaged over sex. Post hoc pairwise comparisons between treatment groups were adjusted for multiple testing using the Holm method.

## 3. Results

### 3.1. Baseline Characteristics of the Study Population

A total of 222 unique patients were included in the study, contributing 258 distinct therapeutic sequences (baseline observations at T0). This discrepancy reflects a subset of patients who switched between DMT categories during the study period, with each new treatment initiation treated as an independent longitudinal entry. The number of available observations decreased progressively over the follow-up period: 258 at T0, 128 at T1, 80 at T2, 43 at T3, 22 at T4, and 7 at T5.

Baseline demographic and clinical characteristics were stratified by treatment type into ME-DMT and HE-DMT groups, as summarized in Table 1. Of the baseline observations, 100 (38.76%) involved patients receiving HE-DMTs and 158 (61.24%) involved patients receiving ME-DMTs. The distribution of individual therapeutic agents within each treatment group is detailed in Supplementary Table S1.

Statistical analysis revealed a significant divergence between the two groups regarding therapy status at treatment initiation (*p* < 0.01). The majority of patients in the HE-DMT group (67%) entered the study following a therapeutic switch, whereas the ME-DMT group was predominantly composed of treatment-naive individuals (74.68%). Demographically, patients initiated on ME-DMTs were significantly older, exhibiting a mean age of 37.72 ± 12.10 years compared to 34.13 ± 10.37 years in the HE-DMT group (*p* = 0.02). No statistically significant difference was observed in the gender distribution between groups (*p* = 0.27), although a female predominance was noted in both the ME-DMT (72.15%) and HE-DMT (65%) populations. Regarding clinical markers of disability, the HE-DMT group presented with significantly higher baseline EDSS scores, with a median of 2 (IQR 1.5) compared to a median of 1.5 (IQR 1) in the ME-DMT group (*p* < 0.01). Disease duration at treatment initiation differed significantly between groups, with a median of 6.0 (IQR 9.0) years in the HE-DMT group compared to 3.0 (IQR 8.75) years in the ME-DMT group (*p* < 0.01). Conversely, serum UA levels at treatment initiation (T0) demonstrated no significant variation between groups, with mean values of 3.97 ± 1.20 mg/dL for ME-DMT and 4.06 ± 1.38 mg/dL for HE-DMT therapies (*p* = 0.59).

### 3.2. Longitudinal Changes in Uric Acid Levels

The longitudinal evolution of serum UA levels was evaluated using a linear mixed-effects model, adjusting for potential confounders including sex, age, and baseline EDSS scores. The results of the linear mixed-effects model, representing the tests of fixed effects, are summarized in Table 2.

The analysis demonstrated that UA levels showed a significant main effect of time over the 5-year follow-up period (F(5, 307.04) = 11.03, *p* < 0.01). Furthermore, a significant main effect of treatment type was observed (F(1, 378.31) = 5.25, *p* = 0.02), alongside a significant interaction between time and treatment group (F(5, 307.05) = 2.42, *p* = 0.04). This interaction confirms that the trajectories of UA changes differed significantly between the HE-DMT and ME-DMT cohorts over time.

In the HE-DMT group, mean UA levels increased from 4.27 mg/dL at baseline to 4.58 mg/dL at year 1 and 4.72 mg/dL at year 2, reaching higher levels at year 3 (5.29 mg/dL) and year 4 (5.58 mg/dL), then remaining elevated at year 5 (5.32 mg/dL).

**Figure 1:**
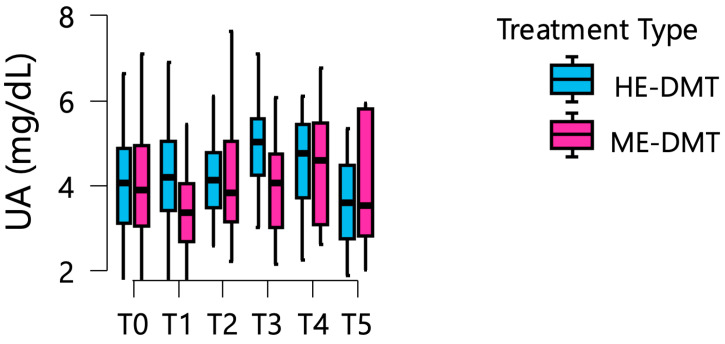
Longitudinal trajectories of serum uric acid levels by treatment group. Box plots display the distribution of uric acid levels at each time point (T0 through T5) stratified by treatment type (HE-DMT in blue vs. ME-DMT in magenta). Boxes represent the interquartile range (IQR, 25th to 75th percentiles), horizontal lines within boxes indicate medians, and whiskers extend to 1.5 × IQR.

In the ME-DMT group, mean UA levels started at 4.19 mg/dL at baseline, decreased to 3.97 mg/dL at year 1, and then increased to 4.54 mg/dL at year 2 and 4.62 mg/dL at year 3, reached 5.04 mg/dL at year 4, and decreased to 4.71 mg/dL at year 5. Comparison of trajectories indicated that the HE-DMT group maintained higher mean UA levels throughout the majority of the study period, most notably during years 1, 3, and 4 (Figure 1). The divergence between groups became apparent as early as year 1 and remained consistent throughout follow-up. Detailed estimated marginal means for key follow-up intervals are presented in Table 3.

### 3.3. Covariate Effects

Regarding the influence of covariates, sex was identified as a significant independent predictor of UA levels (F(1, 210.78) = 68.28, *p* < 0.01). Notably, after adjusting for other model predictors, neither age (F(1, 235.81) = 0.49, *p* = 0.49) nor baseline EDSS score (F(1, 224.62) = 1.03, *p* = 0.31) demonstrated a significant association with UA variation.

Spearman correlation analysis performed at T0 (Table 4) revealed a weak but statistically significant positive correlation between serum UA levels and disease duration (rho = 0.142, *p* = 0.022). No significant correlations were observed between UA levels and age (rho = −0.055, *p* = 0.389) or baseline EDSS (rho = 0.062, *p* = 0.326).

The Mann–Whitney U test (Table 5) confirmed a significant difference in serum UA levels between sexes at treatment initiation (*p* < 0.01). No significant difference in baseline UA levels was observed between treatment-naive patients and switchers at T0 (*p* = 0.236).

### 3.4. Treatment Group Comparison

Final contrast analysis confirmed a statistically significant difference between treatment groups after applying the Holm correction for multiple testing (estimate = 0.53, 95% CI [0.18, 0.87], z = 2.98, *p* < 0.01), specifically highlighting a more pronounced increase in the HE-DMT group during the first year of therapy.

## 4. Discussion

We demonstrated that HE-DMTs are associated with significantly greater increases in serum UA levels compared to ME-DMTs over a 5-year follow-up period. Our study advances current knowledge by extending prior observations on UA alterations in MS into a longitudinal, within-patient framework.

Early case–control studies demonstrated significantly reduced UA concentrations in MS relative to both healthy subjects and patients with other neurological or inflammatory conditions, suggesting a disease-specific rather than nonspecific inflammatory effect [6,7]. These findings have been reinforced by meta-analytic evidence, with Liu et al., 2012, confirming significantly lower UA levels across multiple MS cohorts despite methodological heterogeneity [16]. Reduced UA levels have also been described early in the disease course, including in patients with clinically isolated syndrome, indicating that this alteration may be present from the earliest stages of MS [6]. However, associations between UA levels and clinical severity or radiological burden have been inconsistent. While some studies reported lower UA levels during periods of clinical activity, others did not identify robust correlations with disability progression or MRI measures, underscoring substantial interindividual variability and phenotypic heterogeneity within MS [6,16,17].

By applying longitudinal mixed-effects modeling over an extended follow-up period and stratifying patients according to DMT efficacy, our study provides a novel perspective on how UA levels evolve over time within MS patients under different therapeutic contexts. Using this approach, time-dependent changes in UA levels, treatment efficacy, and their interaction all reached statistical significance, indicating distinct UA trajectories in the HE-DMT and ME-DMT groups. Baseline UA levels at treatment initiation or modification (T0) were comparable between groups despite differences in age, EDSS, treatment status, and disease duration, supporting the interpretation that the subsequent divergence reflected longitudinal changes emerging during follow-up rather than pre-existing biochemical differences. An important aspect to consider is the difference in treatment status at baseline, as patients in the HE-DMT group were more frequently undergoing treatment switching, whereas the ME-DMT group was predominantly treatment-naive. This likely reflects differences in prior disease activity and treatment response, which may have influenced subsequent UA trajectories. Among patients initiating therapy following a treatment switch, no significant difference in baseline serum UA levels was observed between the HE-DMT and ME-DMT groups, suggesting that prior therapeutic exposure did not differentially influence UA levels at treatment initiation. Although disease duration differed significantly between treatment groups, the association between disease duration and serum UA levels at treatment initiation was weak (rho = 0.142), suggesting that disease duration alone did not substantially drive UA variability in our cohort.

During follow-up, HE-DMTs were associated with an earlier and greater increase in UA levels compared with ME-DMT, with the largest separation observed during the first treatment year and persisting across subsequent time points. The temporal pattern observed in this study, with early divergence followed by sustained separation, is biologically plausible in the context of rapid suppression of inflammatory activity under high-efficacy therapies. These observations further support the distinction between UA as a state-dependent rather than a trait biomarker in MS. While baseline levels appear to have limited predictive value, longitudinal changes may better reflect dynamic interactions between inflammatory activity and treatment effects over time.

One way to interpret the efficacy-stratified separation is through the lens of redox balance rather than direct effects on UA metabolism. If circulating UA levels partly reflect the balance between oxidative and nitrosative stress and endogenous antioxidant capacity, then different longitudinal trajectories may correspond to differences in how inflammatory activity is modulated over time. The fact that group separation was most pronounced early after treatment initiation or modification suggests a transition phase during which the systemic oxidative milieu may re-equilibrate. This interpretation is compatible with prior observations indicating that UA behaves as a state-dependent parameter in MS, with lower levels reported during relapse and in association with blood–brain barrier disruption, implying that periods of increased inflammatory activity may coincide with reduced circulating UA and vice versa [16,18].

An alternative interpretation is that UA levels may act as an indirect marker of inflammatory burden rather than a direct mediator of disease processes. In this context, higher UA levels observed under high-efficacy therapies could reflect reduced consumption due to lower oxidative stress rather than increased production.

The absence of baseline UA differences between treatment groups in our cohort, together with genetic and prospective data arguing against a causal role for UA in MS susceptibility, suggests that baseline UA alone is unlikely to inform treatment selection [6,19,20]. In contrast, persistent longitudinal patterns, such as consistently low UA levels or failure to show an upward shift over time, may be more informative than single measurements. This interpretation is supported by longitudinal evidence linking within-patient UA variation to clinically meaningful outcomes, including relapse risk, disability progression, and cognitive performance, underscoring the relevance of dynamic UA assessment rather than static thresholds [8].

Beyond their mechanistic interpretation, the observed longitudinal differences in UA trajectories raise the possibility that serial UA measurements, rather than isolated baseline values, could contribute to monitoring disease evolution under treatment. Given evidence that UA levels vary with disease state, sustained reductions or renewed declines in UA during follow-up could plausibly coincide with periods of increased inflammatory burden and prompt closer clinical or radiological assessment, although such applications would require prospective validation [16,17,18].

If confirmed in prospective studies incorporating concurrent clinical and MRI activity, UA trajectories could therefore be explored as an adjunctive longitudinal signal complementing established measures of disease activity rather than replacing them. At the same time, any framework that considers rising UA levels must balance potential interpretive value with safety considerations. Although available data do not suggest that standard MS DMTs induce UA-related adverse effects, interventional studies aimed at pharmacologically increasing UA have reported complications such as nephrolithiasis, highlighting the need to consider upper-range UA values when interpreting sustained longitudinal increases [21]. Careful longitudinal interpretation would thus need to account for both potential informational value and individual risk profiles.

Several limitations should be considered. First, the retrospective observational design limits causal interpretation of the association between DMT exposure and UA trajectories, and residual confounding by unmeasured factors such as dietary habits, body composition, or metabolic variability cannot be excluded. Second, the lack of concurrent markers of oxidative or nitrosative stress limits mechanistic interpretation of the observed changes in UA levels. The absence of information regarding relapse activity and MRI findings limits the ability to directly correlate UA trajectories with established measures of disease activity. Third, although the 5-year follow-up provides a long-term perspective, the sample size decreased at later time points, as reflected by the larger standard errors at T4 and T5. This dropout is typical of real-world retrospective cohorts but necessitates caution when interpreting the long-term stability of the observed trajectories.

Future prospective studies should integrate concurrent relapse rates, MRI activity, NEDA status, and standardized biochemical markers of oxidative stress, alongside systematic collection of potential confounders such as BMI, dietary habits, and renal function, to clarify the clinical significance and biological determinants of longitudinal UA trajectories in MS.

Taken together, these findings highlight the dynamic nature of UA levels in MS and underscore the need for further prospective evaluation of their longitudinal behavior in relation to disease activity and treatment context.

## 5. Conclusions

In this retrospective longitudinal cohort study, serum UA levels in MS patients showed significant time-dependent variation and differed according to DMT efficacy over extended follow-up. Patients receiving HE-DMTs exhibited a distinct longitudinal trajectory characterized by an earlier and more pronounced increase in UA levels compared with those receiving ME-DMTs. These findings indicate that UA levels in MS are dynamic and evolve over time in relation to treatment context rather than representing a fixed biochemical characteristic. While the clinical implications of these observations require prospective validation linked to clinical and radiological outcomes, the results support further investigation of longitudinal UA trajectories as a complementary indicator of treatment-associated changes in redox balance in MS.

## Figures and Tables

**Table 1:** Baseline Demographic and Clinical Characteristics of the Study Population.

Characteristic	ME-DMT (n = 158)	HE-DMT (n = 100)	*p*-Value
Therapy Status, n (%)			<0.01 *
Treatment-naive	118 (74.68%)	33 (33.00%)	
Switching therapy	40 (25.32%)	67 (67.00%)	
Sex, n (%)			0.27
Female	114 (72.15%)	65 (65.00%)	
Male	44 (27.85%)	35 (35.00%)	
Age (years), mean ± SD	37.72 ± 12.10	34.13 ± 10.37	0.02 *
EDSS score, median [IQR]	1.5 [1.0]	2.0 [1.5]	<0.01 *
Disease duration (years), median [IQR]	3.0 [8.75]	6.0 [9.0]	<0.01 *
Uric acid (mg/dL), mean ± SD	3.97 ± 1.20	4.06 ± 1.38	0.59

Data are presented as n (%), mean ± SD, or median (IQR) as appropriate based on distribution. Statistical comparisons: Fisher’s exact test for categorical variables (treatment status, sex); Mann–Whitney U test for non-normally distributed continuous variables (EDSS, disease duration); independent samples *t*-test for normally distributed continuous variables (age, uric acid). ME-DMT = moderate-efficacy disease-modifying therapies; HE-DMT = high-efficacy disease-modifying therapies; EDSS = Expanded Disability Status Scale; SD = standard deviation; IQR = interquartile range. * Statistically significant at *p* < 0.05.

**Table 2:** Analysis of variance for the linear mixed model of uric acid levels.

Effect	df	F	*p*
Time period	5, 307.04	11.03	<0.01 *
Treatment type (HE-DMT vs. ME-DMT)	1, 378.31	5.25	0.02
Sex	1, 210.78	68.28	<0.01 *
Age	1, 235.81	0.49	0.49
Baseline EDSS	1, 224.62	1.03	0.31
Time × treatment type	5, 307.05	2.42	0.04 *

Model fitted using restricted maximum likelihood (REML) with random intercepts for patients. Degrees of freedom werecalculated using Satterthwaite approximation. Type III sums of squares. HE-DMT = high-efficacy disease-modifying therapies; ME-DMT = moderate-efficacy disease-modifying therapies; EDSS = Expanded Disability Status Scale; df = degrees of freedom; F = F-statistic. * Statistically significant at *p* < 0.05.

**Table 3:** Estimated marginal means of serum uric acid levels by treatment group.

Time	HE-DMT Mean (SE)	HE-DMT 95% CI	ME-DMT Mean (SE)	ME-DMT 95% CI
Baseline (T0)	4.27 (0.12)	[4.03, 4.51]	4.19 (0.10)	[3.99, 4.38]
T1	4.58 (0.13)	[4.32, 4.84]	3.97 (0.14)	[3.69, 4.25]
T2	4.72 (0.16)	[4.41, 5.03]	4.54 (0.16)	[4.23, 4.86]
T3	5.29 (0.22)	[4.86, 5.72]	4.62 (0.19)	[4.25, 4.99]
T4	5.58 (0.34)	[4.91, 6.26]	5.04 (0.23)	[4.59, 5.49]
T5	5.32 (0.57)	[4.20, 6.43]	4.71 (0.38)	[3.96, 5.46]

Values are in mg/dL, adjusted for sex, age, and EDSS. SE = standard error; CI = confidence interval; HE-DMT = high-efficacy disease-modifying therapies; ME-DMT = moderate-efficacy disease-modifying therapies. Larger standard errors at years 4–5 reflect reduced sample sizes.

**Table 4:** Spearman correlation analysis of UA levels at T0.

Variable	Spearman’s Rho	*p*
Age	−0.055	0.389
Disease duration	0.142	0.022 *
EDSS	0.062	0.326

* Statistically significant at *p* < 0.05.

**Table 5:** Mann–Whitney U test comparisons of UA levels at T0.

Comparison	*p*
Male vs. female	<0.01 *
Naive vs. switcher	0.236

* Statistically significant at *p* < 0.05.

## Data Availability

The data are available upon request from the corresponding author. The data are not publicly available due to privacy and ethical restrictions related to patient confidentiality.

## Supplementary Materials

The following supporting information can be downloaded at: https://www.mdpi.com/article/10.3390/medsci14020256/s1, Table S1: Distribution of patients by therapeutic agent and therapy status at treatment initiation.

## Abbreviations

The following abbreviations are used in this manuscript:

BMI	Body Mass Index
CI	Confidence interval
df	Degrees of freedom
DMT	Disease-modifying therapy
EDSS	Expanded Disability Status Scale
HE-DMT	High-efficacy disease-modifying therapy
IQR	Interquartile range
ME-DMT	Moderate-efficacy disease-modifying therapy
MRI	Magnetic resonance imaging
MS	Multiple sclerosis
REML	Restricted maximum likelihood
RRMS	Relapsing-remitting multiple sclerosis
SD	Standard deviation
SE	Standard error
UA	Uric acid

## References

[1] Campbell G.R., Mahad D.J. (2012). Clonal expansion of mitochondrial DNA deletions and the progression of multiple sclerosis. CNS Neurol. Disord. Drug Targets.

[2] Cross A.H., Manning P.T., Keeling R.M., Schmidt R.E., Misko T.P. (1998). Peroxynitrite formation within the central nervous system in active multiple sclerosis. J. Neuroimmunol..

[3] Tanaka M., Vécsei L. (2020). Monitoring the redox status in multiple sclerosis. Biomedicines.

[4] Hooper D.C., Scott G.S., Zborek A., Mikheeva T., Kean R.B., Koprowski H., Spitsin S.V. (2000). Uric acid, a peroxynitrite scavenger, inhibits CNS inflammation, blood-CNS barrier permeability changes, and tissue damage in a mouse model of multiple sclerosis. FASEB J..

[5] Constantinescu C.S., Freitag P., Kappos L. (2000). Increase in serum levels of uric acid, an endogenous antioxidant, under treatment with glatiramer acetate for multiple sclerosis. Mult. Scler..

[6] Rentzos M., Nikolaou C., Anagnostouli M., Rombos A., Tsakanikas K., Economou M., Dimitrakopoulos A., Karouli M., Vassilopoulos D. (2006). Serum uric acid and multiple sclerosis. Clin. Neurol. Neurosurg..

[7] Sotgiu S., Pugliatti M., Sanna A., Sotgiu A., Fois M.L., Arru G., Rosati G. (2002). Serum uric acid and multiple sclerosis. Neurol. Sci..

[8] Moccia M., Lanzillo R., Costabile T., Russo C., Carotenuto A., Sasso G., Postiglione E., De Luca Picione C., Vastola M., Maniscalco G.T. (2015). Uric acid in relapsing-remitting multiple sclerosis: A 2-year longitudinal study. J. Neurol..

[9] Guerrero A.L., Martín-Polo J., Laherrán E., Gutiérrez F., Iglesias F., Tejero M.A., Rodríguez-Gallego M., Alcázar C. (2008). Variation of serum uric acid levels in multiple sclerosis during relapses and immunomodulatory treatment. Eur. J. Neurol..

[10] Thompson A.J., Banwell B.L., Barkhof F., Carroll W.M., Coetzee T., Comi G., Correale J., Fazekas F., Filippi M., Freedman M.S. (2018). Diagnosis of multiple sclerosis: 2017 revisions of the McDonald criteria. Lancet Neurol..

[11] Park S., Chi S., Yang J.H., Min M., Shin J.Y. (2024). Comparison of uric acid elevation between aspirin-ticagrelor and aspirin-clopidogrel during dual antiplatelet therapy. Int. J. Clin. Pharmacol. Ther..

[12] McAdams DeMarco M.A., Maynard J.W., Baer A.N., Gelber A.C., Young J.H., Alonso A., Coresh J. (2012). Diuretic use, increased serum urate levels, and risk of incident gout in a population-based study of adults with hypertension: The Atherosclerosis Risk in Communities cohort study. Arthritis Rheum..

[13] Filippi M., Amato M.P., Centonze D., Gallo P., Gasperini C., Inglese M., Patti F., Pozzilli C., Preziosa P., Trojano M. (2025). The use of high-efficacy disease-modifying therapies in multiple sclerosis: Recommendations from an expert Delphi consensus. J. Neurol..

[14] Drulović J., Dujmović I., Stojsavljević N., Mesaros S., Andjelković S., Miljković D., Perić V., Dragutinović G., Marinković J., Lević Z. (2001). Uric acid levels in sera from patients with multiple sclerosis. J. Neurol..

[15] Rabe-Hesketh S., Skrondal A. (2012). Multilevel and Longitudinal Modeling Using Stata.

[16] Liu B., Shen Y., Xiao K., Tang Y., Cen L., Wei J. (2012). Serum uric acid levels in patients with multiple sclerosis: A meta-analysis. Neurol. Res..

[17] Toncev G., Milicic B., Toncev S., Samardzic G. (2002). Serum uric acid levels in multiple sclerosis patients correlate with activity of disease and blood-brain barrier dysfunction. Eur. J. Neurol..

[18] Guerrero A.L., Gutiérrez F., Iglesias F., Martín-Polo J., Merino S., Martín-Serradilla J.I., Laherrán E., Tejero M.A. (2011). Serum uric acid levels in multiple sclerosis patients inversely correlate with disability. Neurol. Sci..

[19] Massa J., O’Reilly E., Munger K.L., Delorenze G.N., Ascherio A. (2009). Serum uric acid and risk of multiple sclerosis. J. Neurol..

[20] Niu P.P., Song B., Wang X., Xu Y.M. (2020). Serum uric acid level and multiple sclerosis: A Mendelian randomization study. Front. Genet..

[21] Markowitz C.E., Spitsin S., Zimmerman V., Jacobs D., Udupa J.K., Hooper D.C., Koprowski H. (2009). The treatment of multiple sclerosis with inosine. J. Altern. Complement. Med..

