# Supplementary material for: Serum Uric Acid Trajectories in Multiple Sclerosis: A 5-Year Longitudinal Comparison of High- and Moderate-Efficacy Therapies
Source: Med Sci (Basel). 2026 May 15;14(2):256. doi: 10.3390/medsci14020256 (PMC13214808; doi:10.3390/medsci14020256)
Supplement: Supplementary file 1 [file medsci-14-00256-s001.zip › medsci-4270404-supplementary.pdf]

Table S1. Distribution of patients by therapeutic agent and therapy status at treatment initiation.

**Agent**	**Naive n (%)**	**Switch n (%)**	**Total n (%)**
Interferon β-1a (Avonex)	15 (83.3%)	3 (16.7%)	18 (7.0%)
Interferon β-1b (Betaferon)	14 (87.5%)	2 (12.5%)	16 (6.2%)
Interferon β-1a (Rebif)	32 (100%)	-	32 (12.4%)
Glatiramer acetate	12 (80.0%)	3 (20.0%)	15 (5.8%)
Teriflunomide	26 (53.1%)	23 (46.9%)	49 (19.0%)
Dimethyl fumarate	17 (65.4%)	9 (34.6%)	26 (10.1%)
Plegridy	2 (100%)	-	2 (0.8%)
**ME-DMT Total**	**118 (74.7%)**	**40 (25.3%)**	**158 (61.2%)**
Natalizumab	13 (34.2%)	25 (65.8%)	38 (14.7%)
Ocrelizumab	12 (27.9%)	31 (72.1%)	43 (16.7%)
Cladribine	7 (43.8%)	9 (56.3%)	16 (6.2%)
Fingolimod	-	1 (100%)	1 (0.4%)
Ofatumumab	1 (100%)	-	1 (0.4%)
Alemtuzumab	-	1 (100%)	1 (0.4%)
**HE-DMT Total**	**33 (33.0%)**	**67 (67.0%)**	**100 (38.8%)**
**Total**	**151 (58.5%)**	**107 (41.5%)**	**258 (100%)**
